# Spontaneous Rupture of a Peritonsillar Abscess Mimicking Successful Incision and Drainage: A Case Report

**DOI:** 10.7759/cureus.108365

**Published:** 2026-05-06

**Authors:** Roda Rashid Mohamed Bin S Alshamsi, Sawsan Horani, Fatima Sami Alhammadi, Tarek Al Salhani

**Affiliations:** 1 Graduate Medical Education, Mohammed Bin Rashid University of Medicine and Health Sciences, Dubai Health, Dubai, ARE; 2 Otolaryngology - Head and Neck Surgery, Dubai Health, Dubai, ARE

**Keywords:** deep neck space infection, hot potato voice, peritonsillar abscess, spontaneous abscess rupture, trismus

## Abstract

Peritonsillar abscess (PTA) is a common deep infection of the head and neck that can present with significant clinical symptoms. Patients typically present with fever, malaise, sore throat, otalgia, dysphagia, and the characteristic muffled “hot-potato” voice. Although PTA is generally managed effectively with antibiotics, analgesics, and abscess drainage, spontaneous rupture is uncommon and may falsely suggest clinical resolution, posing a diagnostic and management challenge. We present the case of a 25-year-old woman who presented with a five-day history of sore throat, fever, odynophagia, dysphagia, hot-potato voice, and foul taste in the mouth. Examination revealed right peritonsillar swelling with a mucosal opening consistent with spontaneous rupture of the abscess. The rupture was presumed to have occurred prior to presentation and was further supported by the patient’s history of a sudden onset of a foul taste around the third day of symptoms, followed by partial symptomatic relief. Laboratory investigations demonstrated significant inflammation, with a C-reactive protein (CRP) level of 190 mg/L and neutrophilic leukocytosis. The patient was admitted for intravenous antibiotics, dexamethasone, and observation; however, she requested discharge against medical advice one day after admission due to reported symptomatic improvement and travel plans. Spontaneous rupture of a peritonsillar abscess is a rare event that may lead to transient symptom relief and a misleading impression of recovery due to possible incomplete drainage. This case highlights the importance of careful clinical evaluation and continued management despite apparent improvement, as persistent infection and complications may still occur.

## Introduction

Peritonsillar abscess (PTA) remains the most common deep neck space infections of the head and neck across all age groups, although it is most frequently encountered in young adults and is usually a complication of acute tonsillitis [1]. The reported annual incidence ranges from approximately 10 to 37 cases per 100,000 population, with studies from the United States estimating approximately 30 cases per 100,000 persons annually [1,2]. However, epidemiological data from the United Arab Emirates remain limited.

It mainly affects adults between 20 and 40 years of age but can occur in any age group [1,3]. Patients with PTA typically present to the emergency department acutely ill and report unilateral severe throat pain as the main presenting complaint. Associated symptoms include high-grade fever, malaise, referred otalgia, dysphagia, odynophagia, trismus, muffled “hot-potato” voice, and drooling [1,4]. 

Clinical signs include characteristic peritonsillar swelling with associated oropharyngeal asymmetry, soft palate swelling, uvular deviation, and cervical lymphadenitis [1,4]. If left untreated, infection can progress into other deep neck planes, developing sepsis and life-threatening complications such as airway obstruction, descending mediastinitis, septic thrombophlebitis, or hemorrhage [4,5].

The microbiology of PTA is typically polymicrobial, involving aerobic and anaerobic organisms derived from the normal oral flora [5]. Common pathogens include *Streptococcus* species alongside anaerobic bacteria such as *Fusobacterium* and *Prevotella* [1,5]

While the presentation and management of PTA are well established, spontaneous rupture of PTA remains poorly described in the literature [6,7]. Although no dedicated clinical practice guideline exists specifically for PTA, current otolaryngology practice supports prompt drainage of the abscess through needle aspiration or incision and drainage in conjunction with appropriate antibiotic therapy and supportive care [1,8].

Unlike controlled incision and drainage, spontaneous rupture can cause a rapid decompression of the abscess cavity and consequently rapid relief of symptoms, which may create a misleading impression of clinical resolution. However, incomplete drainage may result in persistent infection and may increase the risk of extension into other deep neck spaces [5,9]. As the literature on spontaneous rupture remains limited and largely consists of isolated case reports, its true incidence, clinical course, management strategies, and complication risk remain poorly defined.

The clinical significance of spontaneous drainage of PTA lies in its deceptive presentation, the limited visual documentation of the phenomenon, and the conservative management that follows. Although patients may experience symptomatic relief following rupture, underlying inflammation and infection may persist, potentially delaying appropriate treatment [10]. In addition, complications such as aspiration, airway compromise, and spread to deep neck spaces have been reported and, although rare, may lead to serious outcomes [5,11].

This case is presented to highlight spontaneous rupture as an atypical and clinically confusing presentation of PTA, emphasizing the need for continued vigilance and appropriate management despite apparent symptomatic improvement [7].

## Case presentation

A 25-year-old female patient presented to the emergency department with a five-day history of sore throat. This was associated with fever, diaphoresis, odynophagia, dysphagia with poor oral intake, generalized body aches, and a voice change. She also reported swallowing a foul, metallic-tasting fluid two to three days prior to presentation. She denied shortness of breath and chest pain. Her past medical, surgical, and family history were unremarkable. The patient had recently traveled from Turkey one week prior to admission.

**Figure 1:**
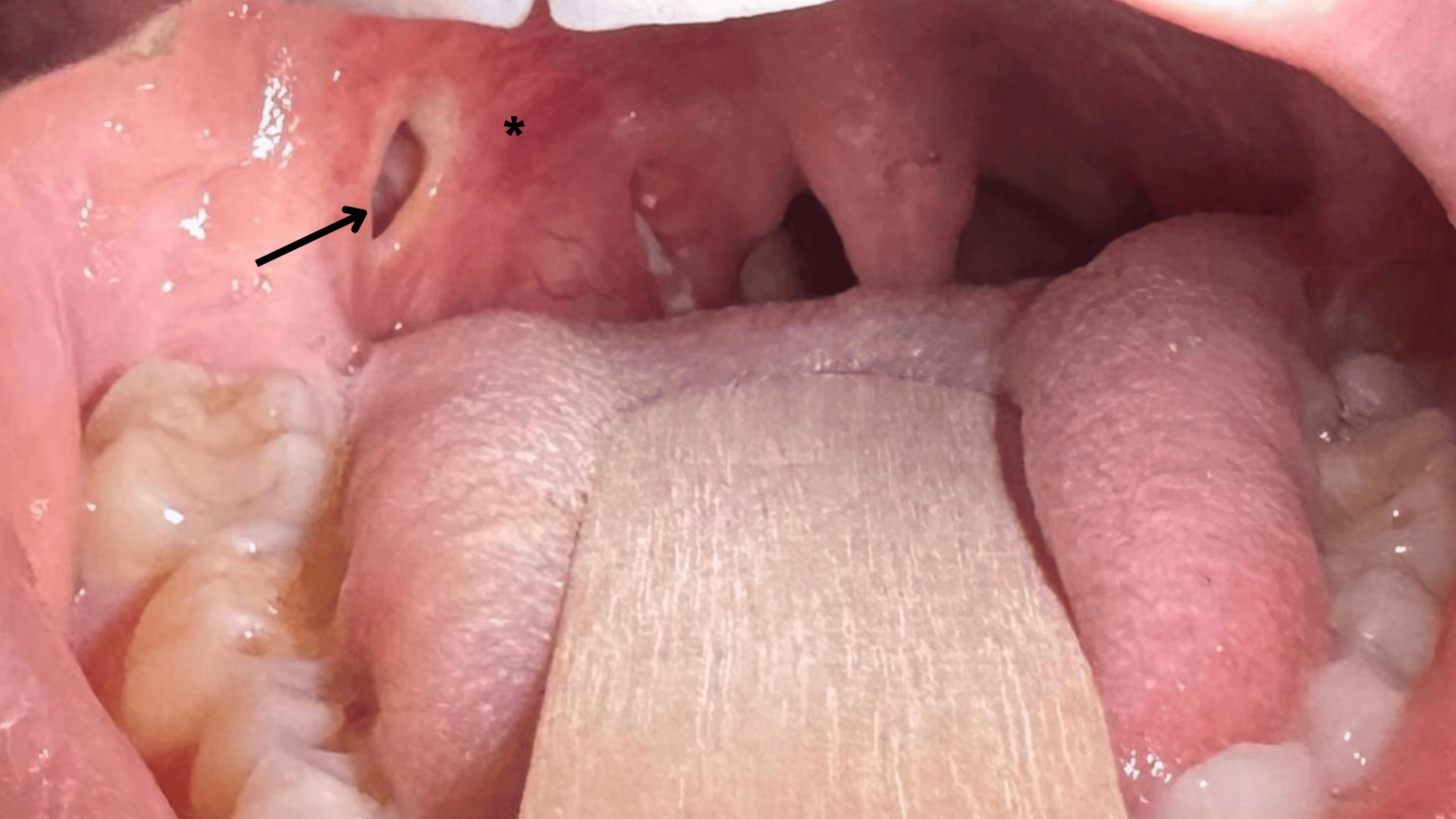
Oropharyngeal photograph demonstrating right peritonsillar swelling with a visible mucosal opening consistent with spontaneous abscess rupture (arrow) and surrounding erythema (*). The uvula appears midline.

On examination, the patient was afebrile and hemodynamically stable (blood pressure 120/79 mmHg, pulse 98/min, temperature 36.7 °C, saturation of peripheral oxygen (SpO₂) 98%). Oropharyngeal examination revealed right peritonsillar edema and erythema, a mucosal opening leading to a right peritonsillar abscess cavity, and trismus with mouth opening only two-finger breadths wide. The uvula was midline. Moreover, cervical tenderness was more pronounced on the right side, with palpable cervical lymphadenopathy (Figure 1).

During assessment, spontaneous rupture of the peritonsillar abscess with residual minimal purulent discharge was noted, after which the patient reported partial symptomatic relief. Laboratory investigations demonstrated markedly elevated C-reactive protein (CRP) and leukocytosis with neutrophilia, while renal function remained normal (Table 1). A throat culture was also obtained and showed no growth.

**Table 1:** Laboratory Findings on Admission CRP: C-reactive protein; WBC: white blood cell count

Parameter	Result	Reference Range
CRP	190 mg/L	<5 mg/L
WBC	15.1×10³/µL	4-10×10³/µL
Neutrophils	79.6%	40-70%
Hemoglobin	11.9 g/dL	12-16 g/dL

The patient was admitted for intravenous antibiotics (cefuroxime and metronidazole), dexamethasone, pain control, and observation. A contrast-enhanced neck computed tomography (CT) scan was scheduled to evaluate for possible deep neck space extension, particularly parapharyngeal space involvement. By the first day of admission, her pain had improved and oral intake was adequate. The patient was advised to continue intravenous therapy; however, she declined the CT scan and elected to leave the hospital against medical advice, thereby discontinuing treatment. She was discharged in a stable condition on oral antibiotics and analgesics, with instructions for outpatient follow-up in one week and to return immediately if symptoms worsened. Unfortunately, she was lost to follow-up as she had to travel back to her home country.

## Discussion

**Figure 2:**
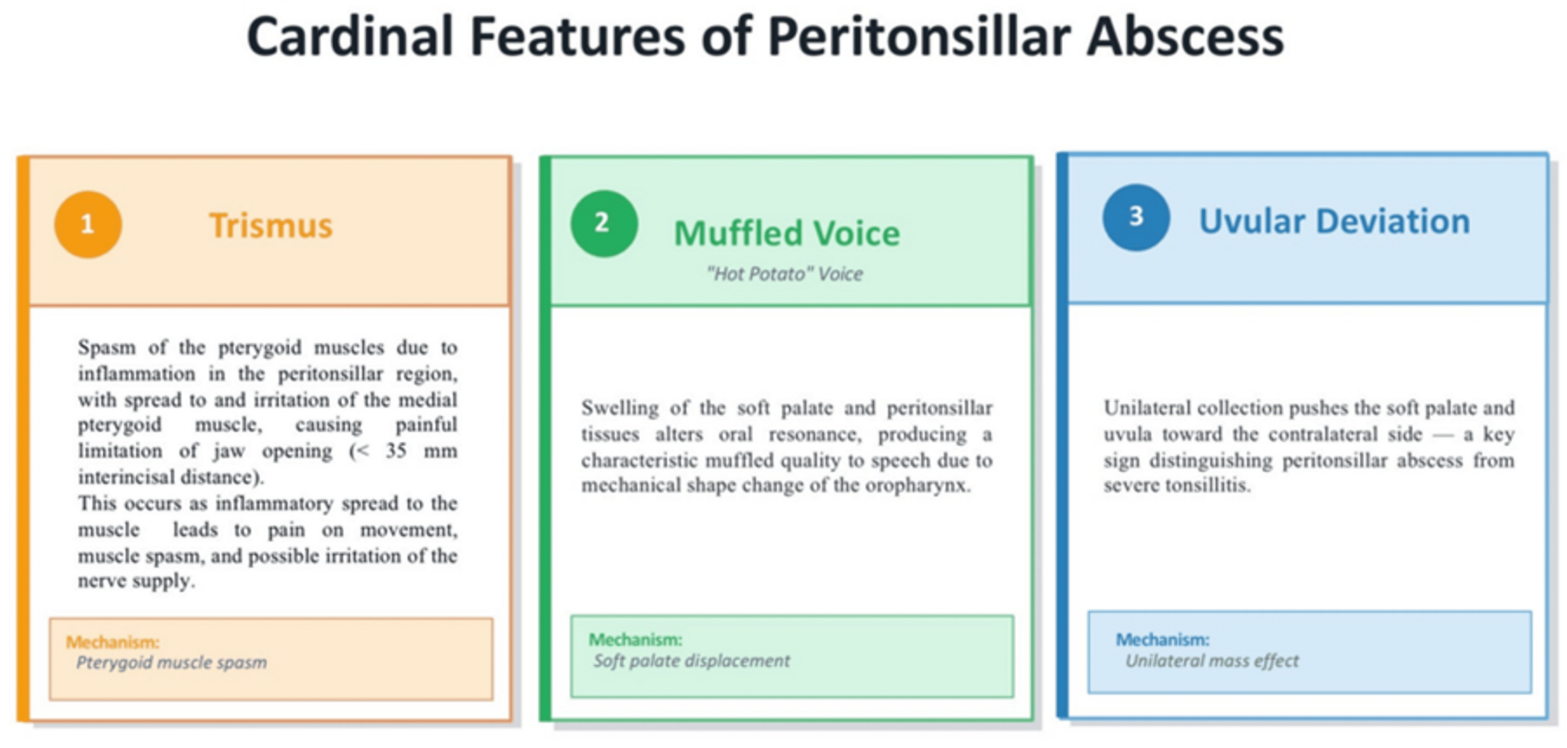
Cardinal clinical features of peritonsillar abscess Illustration created using Microsoft PowerPoint (Microsoft, Redmond, WA).

PTA typically presents with trismus, muffled “hot potato” voice, and uvular deviation (Figure 2).

These findings remain clinically relevant, reflecting the underlying peritonsillar inflammation and mass effect.

In contrast, spontaneous rupture of PTA is rarely reported in the literature, with only a few documented cases available. Most studies elaborate on standard drainage protocols rather than discussing spontaneous rupture of PTA [6]. Various complications have been described following spontaneous rupture, including aspiration pneumonia, persistence of the abscess cavity due to incomplete drainage, and extension into deep neck spaces [5]. Conversely, there is a lack of comparative studies that could evaluate the outcomes of spontaneous rupture against controlled drainage [12]. The limited literature in this area highlights the importance of documenting such cases to improve understanding of the clinical behavior and potential risk patterns associated with spontaneous rupture [7].

**Figure 3:**
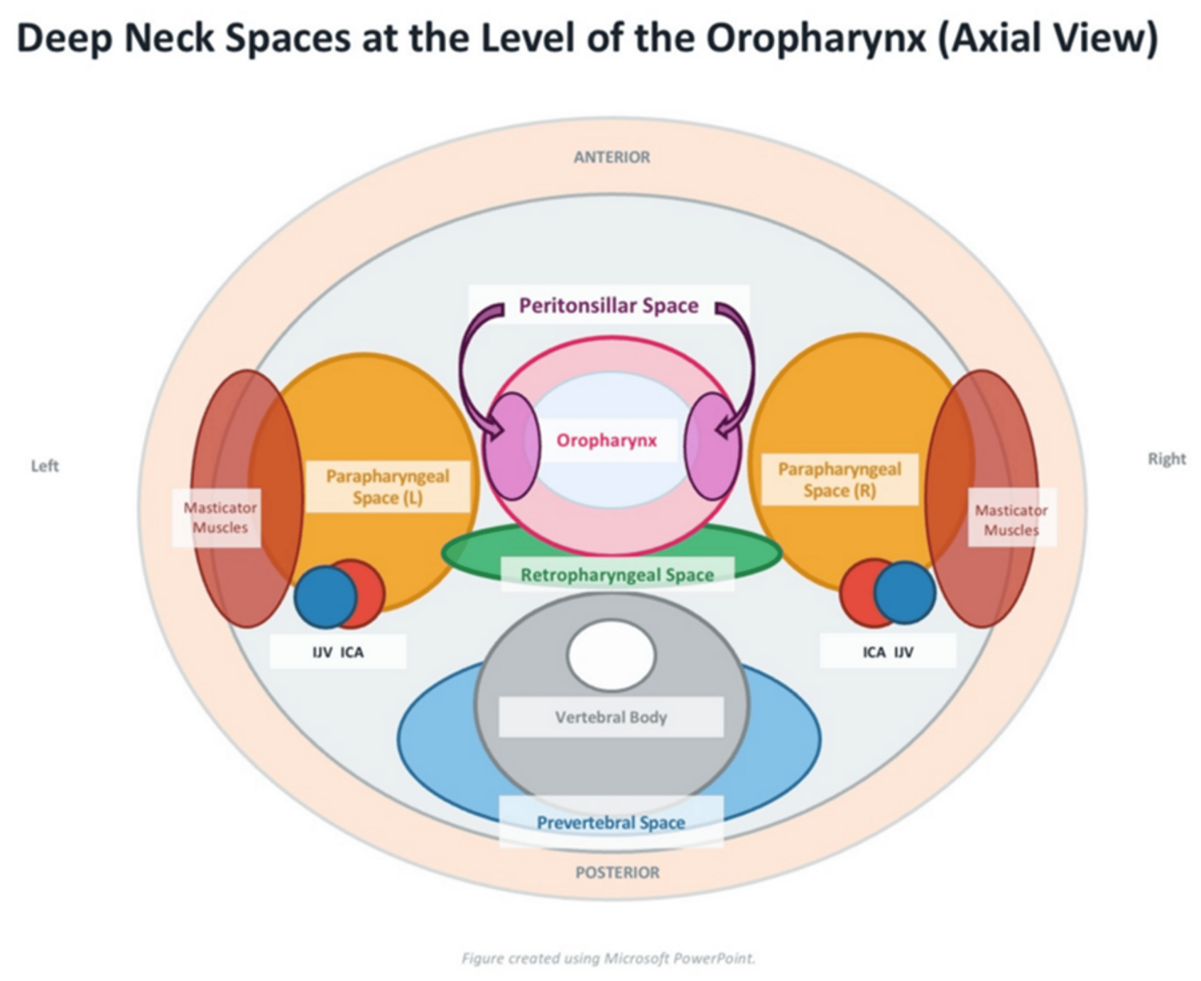
Schematic axial illustration of the deep neck spaces at the level of the oropharynx, demonstrating the anatomical relationships and potential pathways of infection spread from the peritonsillar region to the parapharyngeal and retropharyngeal spaces. Illustration created using Microsoft PowerPoint (Microsoft, Redmond, WA).

Spontaneous rupture is uncommon and differs from controlled incision and drainage in that it may not result in complete evacuation of the abscess cavity. Although it may give a false impression of recovery due to improvement in pain, trismus, and swallowing function following sudden decompression [4], residual loculations and ongoing peritonsillar cellulitis may persist and allow further progression of infection. This distinction is important, as PTA can become complicated and the infection may spread beyond the peritonsillar space. Such complications have been reported in the literature, with spread to cervical planes causing parapharyngeal and retropharyngeal abscesses (Figure 3), descending mediastinitis, necrotizing fasciitis, and other life-threatening sequelae.

This suggests that PTA is not as straightforward as it may initially appear and highlights the need for frequent objective assessment during recovery [5]. Therefore, clinical decisions following spontaneous rupture should be guided by objective markers and reassessment rather than relying solely on subjective improvement [5,13]. Moreover, spontaneous rupture can introduce risks that are not commonly highlighted in the standard discussion of PTA. Pediatric literature has documented cases in which spontaneous rupture led to aspiration pneumonia, highlighting the potential risk to the respiratory system [14]. It may also compromise the airway due to edema, deep neck space extension, or rapid clinical deterioration, thereby increasing the risk of morbidity. These risks can only be mitigated through careful assessment and reliance on objective clinical findings [9,15]. Following spontaneous rupture, the presence of red flag symptoms such as drooling, voice change, neck swelling, worsening trismus or odynophagia, recurrent or persistent fever, or any signs of airway compromise should prompt urgent reassessment for possible deep neck space extension. 

There are several possible pathophysiological pathways that may explain why spontaneous rupture should not be considered curative. First, progressive accumulation of intralesional pus increases pressure within the inflamed mucosa, eventually leading to rupture and sudden decompression. Although this may appear to improve symptoms, complete evacuation of the abscess cavity cannot be guaranteed. Second, significant local inflammation leads to microvascular changes, tissue ischemia, and subsequent necrosis, resulting in weakening of the abscess wall and predisposing it to spontaneous perforation. Lastly, mechanical triggers such as swallowing, coughing, or retching may precipitate rupture and potentially lead to the formation of an abnormal fistulous tract, reinforcing that rupture reflects tissue breakdown rather than controlled resolution [16]. These mechanisms explain why spontaneous rupture does not ensure complete evacuation of the abscess cavity and should not be considered definitive treatment, as residual infection may persist and progress despite transient symptomatic improvement.

Despite spontaneous rupture, the management strategy remains unchanged and includes adequate antimicrobial therapy, analgesia, hydration, and ensuring effective drainage when indicated [17]. 

**Figure 4:**
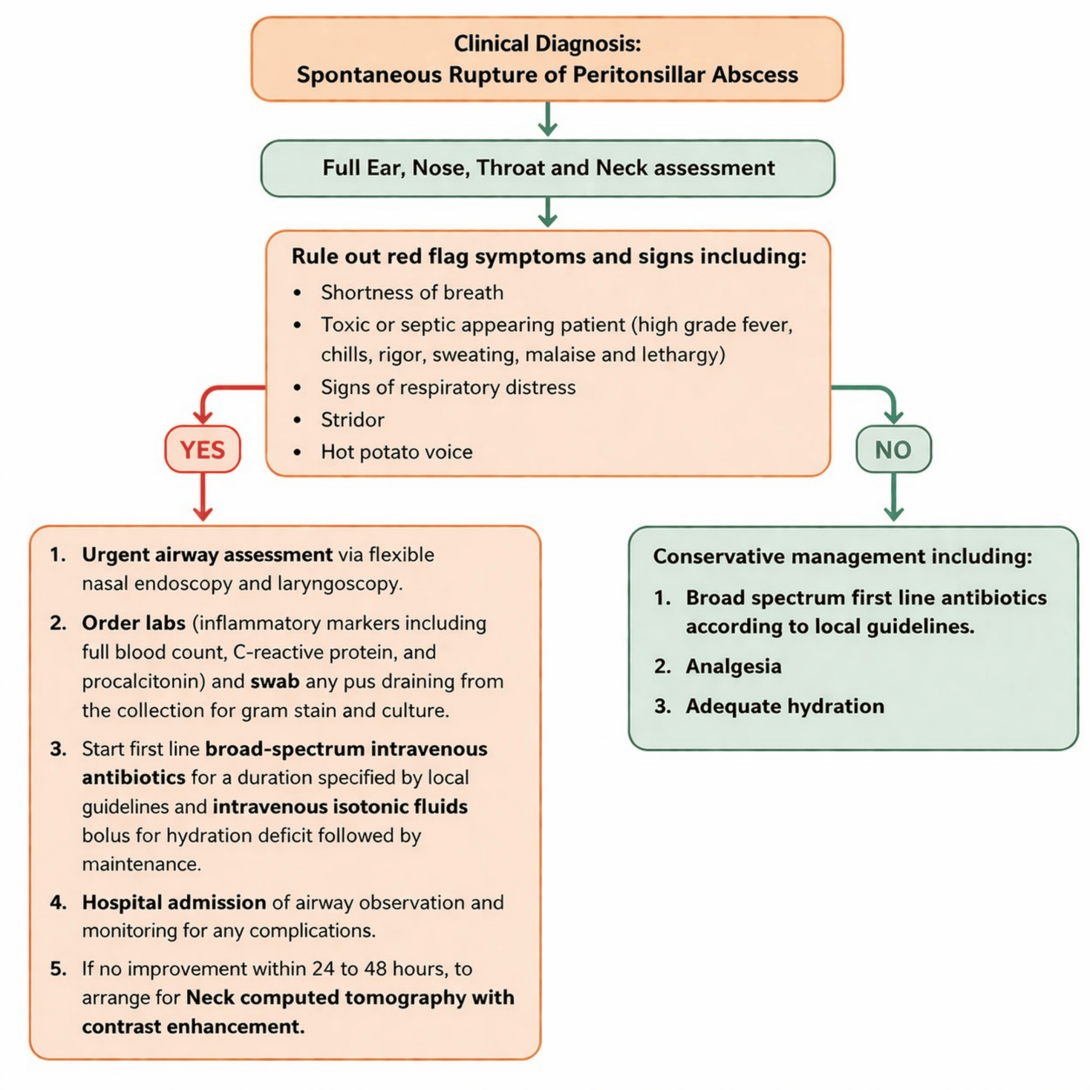
Flowchart of the diagnostic and management approach following spontaneous rupture of peritonsillar abscess Illustration created using Microsoft PowerPoint (Microsoft, Redmond, WA).

A structured approach to the assessment and management of patients following spontaneous rupture is summarized in Figure 4. This framework emphasizes comprehensive Ear, Nose, and Throat (ENT) assessment, identification of red flag symptoms, conservative management in clinically stable patients, and escalation to airway evaluation, laboratory investigation, imaging, and inpatient monitoring when concerning features or inadequate clinical improvement are present. In patients initially managed conservatively, microbiological sampling of purulent drainage may further support pathogen identification and antibiotic optimization when feasible, while contrast-enhanced CT should be guided by clinical progression, persistent symptoms, or suspicion of deeper neck space extension.

Furthermore, patients should be monitored for recurrence and possible complications involving the deep neck spaces, as complete evacuation of the abscess cannot be guaranteed in such cases [5,7]. This becomes even more critical in situations where treatment is discontinued prematurely, such as when a patient leaves the hospital against medical advice. In these situations, careful counseling and clear follow-up instructions are essential. Patients should be warned about the possibility of recurrence or worsening symptoms and advised to seek urgent care if they develop fever, increasing throat pain, worsening dysphagia, drooling, neck swelling, voice change, or breathing difficulty [1,3,4]. Early review is also important to ensure resolution of infection and reduce the risk of relapse or progression.

Additionally, the patient’s decision to leave against medical advice further complicated management, highlighting the importance of patient education and close follow-up in atypical PTA presentations. Leaving against medical advice is associated with higher rates of unplanned readmission and worse short-term outcomes; therefore, patients should be counseled that symptom relief after spontaneous rupture does not equate to cure [11,18]. Accordingly, early review in the ENT clinic is essential to help reduce the risk of reinfection and deep neck space progression [5].

This case report has several limitations. First, it represents a single-patient observation, limiting generalizability. Second, a contrast-enhanced CT scan was planned but not performed due to the patient leaving against medical advice, preventing definitive assessment of deep neck space involvement. Lastly, the patient was lost to follow-up, restricting the evaluation of clinical progression, treatment response, and long-term outcomes.

## Conclusions

Spontaneous rupture of a peritonsillar abscess is a rare event that may obscure persistent infection. This case highlights that apparent clinical improvement following rupture should not replace appropriate antimicrobial therapy, clinical reassessment, and monitoring. Clinicians should remain cautious, as incomplete evacuation of the abscess cavity may allow ongoing infection or progression to deeper neck space complications. Awareness of this atypical presentation is important to avoid under-treatment and ensure timely follow-up and appropriate management.
